# Psychosocial risk factors for suicidality in children and adolescents

**DOI:** 10.1007/s00787-018-01270-9

**Published:** 2019-01-25

**Authors:** J. J. Carballo, C. Llorente, L. Kehrmann, I. Flamarique, A. Zuddas, D. Purper-Ouakil, P. J. Hoekstra, D. Coghill, U. M. E. Schulze, R. W. Dittmann, J. K. Buitelaar, J. Castro-Fornieles, K. Lievesley, Paramala Santosh, C. Arango, the STOP Consortium, Alastair Sutcliffe, Sarah Curran, Laura Selema, Robert Flanagan, Ian Craig, Nathan Parnell, Keren Yeboah, Regina Sala, Jatinder Singh, Federico Fiori, Florence Pupier, Loes Vinkenvleugel, Jeffrey Glennon, Mireille Bakker, Cora Drent, Elly Bloem, Mark-Peter Steenhuis, Ruth Berg, Alexander Häge, Mahmud Ben Dau, Konstantin Mechler, Sylke Rauscher, Sonja Aslan, Simon Schlanser, Ferdinand Keller, Alexander Schneider, Paul Plener, Jörg M. Fegert, Jacqui Paton, Murray Macey, Noha Iessa, Kolozsvari Alfred, Furse Helen, Penkov Nick, Claire Baillon, Hugo Peyre, David Cohen, Olivier Bonnot, Julie Brunelle, Nathalie Franc, Pierre Raysse, Véronique Humbertclaude, Alberto Rodriguez-Quiroga, Covadonga Martínez Díaz-Caneja, Ana Espliego, Jessica Merchán, Cecilia Tapia, Immaculada Baeza, Soledad Romero, Amalia La Fuente, Ana Ortiz, Manuela Pintor, Franca Ligas, Francesca Micol Cera, Roberta Frongia, Bruno Falissard, Ameli Schwalber, Juliane Dittrich, Andrea Wohner, Katrin Zimmermann, Andrea Schwalber, Katherine Aitchison

**Affiliations:** 1 Child and Adolescent Psychiatry Department, Hospital General Universitario Gregorio Marañón, CIBERSAM, Instituto de Investigación Sanitaria Gregorio Marañón (IiSGM), School of Medicine, Universidad Complutense, Madrid, Spain; 2 Child and Adolescent Psychiatry and Psychology Department, 2014SGR489, Institute Clinic of Neurosciences, Hospital Clinic of Barcelona, CIBERSAM, Barcelona, Spain; 3 Child and Adolescent Neuropsychiatry Unit, Department of Biomedical Sciences, University of Cagliari, and “A. Cao” Paediatric Hospital, “G. Brotzu” Hospital Trust, Cagliari University Hospital, Cagliari, Italy; 4 CHRU Montpellier, Hôpital Saint Eloi, Médecine Psychologique de l’Enfant et de l’Adolescent, Montpellier, France; 5 Department of Child and Adolescent Psychiatry, University of Groningen, University Medical Center Groningen, Groningen, The Netherlands; 6 Department of Paediatrics, School of Medicine, Dentistry and Health Sciences, University of Melbourne, Melbourne, Australia; 7 Department of Psychiatry, School of Medicine, Dentistry and Health Sciences, University of Melbourne, Melbourne, Australia; 8 Murdoch Children’s Research Institute, Melbourne, Australia; 9 Division of Neuroscience, School of Medicine, University of Dundee, Dundee, UK; 10 Department of Child and Adolescent Psychiatry/Psychotherapy, University of Ulm, Ulm, Germany; 11 Paediatric Psychopharmacology, Department of Child and Adolescent Psychiatry, Central Institute of Mental Health (CIMH), Medical Faculty Mannheim, University of Heidelberg, Mannheim, Germany; 12 Department of Cognitive Neuroscience, Donders Institute for Brain, Cognition and Behaviour, Radboud University Medical Centre, and Karakter Child and Adolescent Psychiatry University Centre, Nijmegen, The Netherlands; 13 Centro de Investigación Biomédica en Red de Salud Mental, CIBERSAM, Barcelona, Spain; 14 Department of Psychiatry and Clinical Psychology, University of Barcelona, Barcelona, Spain; 15 Department of Child and Adolescent Psychiatry, Institute of Psychology, Psychiatry and Neuroscience, King’s College London, London, UK; 16 Centre for Interventional Paediatric Psychopharmacology and Rare Diseases (CIPPRD), South London and Maudsley NHS Foundation Trust, London, UK; 17 HealthTracker Ltd, Gillingham, Kent UK

**Keywords:** Children, Adolescents, Youth, Suicidality, Risk, Resilience, Psychosocial, Web-based, Questionnaire

## Abstract

Suicidality in childhood and adolescence is of increasing concern. The aim of this paper was to review the published literature identifying key psychosocial risk factors for suicidality in the paediatric population. A systematic two-step search was carried out following the PRISMA statement guidelines, using the terms ‘suicidality, suicide, and self-harm’ combined with terms ‘infant, child, adolescent’ according to the US National Library of Medicine and the National Institutes of Health classification of ages. Forty-four studies were included in the qualitative synthesis. The review identified three main factors that appear to increase the risk of suicidality: psychological factors (depression, anxiety, previous suicide attempt, drug and alcohol use, and other comorbid psychiatric disorders); stressful life events (family problems and peer conflicts); and personality traits (such as neuroticism and impulsivity). The evidence highlights the complexity of suicidality and points towards an interaction of factors contributing to suicidal behaviour. More information is needed to understand the complex relationship between risk factors for suicidality. Prospective studies with adequate sample sizes are needed to investigate these multiple variables of risk concurrently and over time.

## Introduction

Suicide is one of the major causes of death worldwide, and approximately one million people commit suicide each year [1]. The incidence of suicide attempts peaks during the mid-adolescent years, and suicide mortality, which increases with age steadily through the teenage years, is the third leading cause of death in young people between the ages of 10 and 24 [2].

Suicidal acts and behaviours are a matter of great concern for clinicians who deal with paediatric patients with mental health problems. Despite its importance, research on suicidality among children and adolescents has been hampered by the lack of clarity of definition. Beyond suicidal ideation and suicide plans, there are a number of behaviours in which there is an intention to die, including suicide attempts, interrupted attempts, aborted attempts, and other suicidal preparatory acts. Suicidal behaviours require, not only the self-injurious act, but also there must be a suicidal intent. By contrast, when individuals engage in self-injurious behaviours for reasons other than ending their lives, this behaviour is termed non-suicidal self-injury. Deliberate self-harm behaviours comprise self-injurious behaviours regardless their intentionality.

The features of suicidality in children and adolescents are different from those occurring in adults [3] and there is a need for tools to identify those young people at higher risk. Depression is a factor strongly associated with suicidality in this population [4], but it is not present in all cases [5], indicating that suicidal behaviour is a result of the interaction of multiple factors. Furthermore, not all depressed children and adolescents develop suicidal ideation or behaviour [6], indicating the importance of, e.g. social and temperamental factors. Predicting which adolescents are likely to repeat their suicidal behaviour would help to establish prevention and intervention strategies for suicidality in children and adolescents.

Biological, psychological, and social factors contribute to a risk profile in children and adolescents. However, the specific purpose of this paper is to review the literature focusing on psychosocial risk factors and suicidality among children and adolescents.

## Methods

### Search strategy

A systematic two-step search was carried out following the PRISMA statement guidelines [7]. A PubMed search was performed using the following terms: (suicidality, suicide, and self-harm), combined with (infant, child, adolescent) according to the US National Library of Medicine and the National Institutes of Health classification of ages using the filters (humans, clinical trial, randomized controlled trial, English), and limiting the search up to December 2016. This search detected 710 papers. In a second step, the references found in the relevant papers were reviewed, identifying 8 additional publications that had not emerged in the initial search.

### Selection criteria

**Fig. 1:**
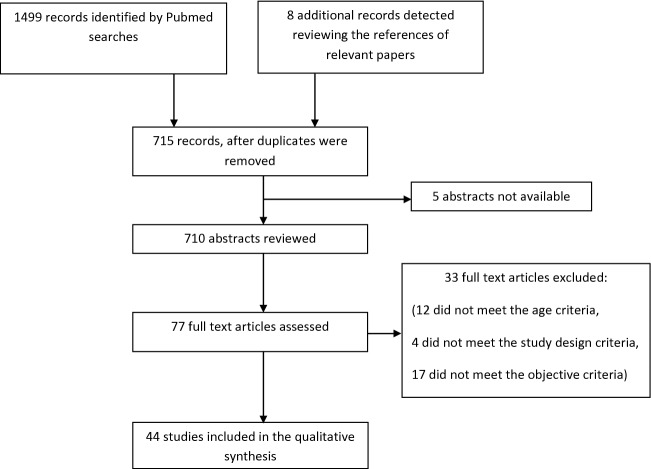
Study selection flowchart (using PRISMA guidelines) [7]

Three researchers (JJC, CL, LK) independently evaluated the abstracts of the 710 studies (see Fig. 1 for flowchart of the literature review). Definitions of suicidal behaviour have varied over time and sometimes differ between the US and Europe. For this review, we considered suicidality a continuum and we used the broader definition of the term self-harm (which includes both suicidal and non-suicidal self-injurious behaviour as described at the Introduction section).

Papers were selected when they met the following criteria:

- Original articles published in English language from initial online databases until December 2016.
- Child and adolescent participants (under 18 years of age). In publications that included adults, only those that reported on children or adolescents separately were considered.
- Publications whose main aim was to examine risk factors for suicidal behaviour/ideation or that included psychosocial variables as risk factors.

Papers were excluded as follows:

- Reviews, editorials, letters, meta-analyses, and guidelines were not considered for this review.
- Studies that investigated the benefit of a therapy (pharmacological, psychotherapeutic, or community intervention), or only analysed suicidal methods, or evaluated psychometric properties of assessment instruments, were excluded.

As a result of this selection process, 77 full-text articles were further assessed.

### Data extraction

The same three researchers (JJC, CL, and LK) reviewed the selected manuscripts. For each study, the following data were extracted: author names, year of publication, number of subjects, age of subjects, inclusion criteria, methodology, and outcome measures.

### Data synthesis and analysis

Studies were classified according to the type of risk factors assessed (psychological factors, adverse life events, and temperament and character factors) and as to sample recruited (clinical vs non clinical samples). Adjusted results were presented.

## Results

### Psychological factors

Twenty-five of the papers reviewed focused on psychological issues as a key outcome measure, and we summarize them below. Depression, previous suicidal attempts, and substance abuse were embedded within a large proportion of the reviewed literature, so we present the studies grouped accordingly. These 25 studies are listed in Tables 1 and 2 (reporting studies based on clinical and non-clinical samples, separately).

**Table 1:** Clinical variables and psychological factors. Clinical samples

References	Sample	Type of study	Measures	Results
Buhren et al. [26]	*N* = 148 Age (mean): 15.2 yr. IC: first onset of anorexia nervosa	Cross-sectional study	BDI EDI-2 K-SADS SIAB-EX	The binge-purging subtype was associated with suicidal ideation (*p* = 0.0008) and self-injurious behaviour (*p* = 0.01)
Brent et al. [18]	*N* = 334 Age: 12–18 yr. IC: CDRS-R ≥ 40 and CGI-S ≥ 4	Prospective study	BDI BHS CBQ C-CASA CDRS-R K-SADS SIQ-Jr	Predictors of suicidal adverse events included self-rated suicidal ideation (OR 1.02, 95% CI 1.01–1.04) and drug or alcohol use (OR 1.9, 95% CI 0.9–3.9) History of non-suicidal self-injury (OR 9.6, 95% CI 3.5–26.1) predicts non-suicidal self-injury events
Vitiello et al. [12]	*N* = 439 Age: 12–17 yr. IC: major depressive disorder	Prospective study	BHS CBQ C-CASA CDRS-R K-SADS-PL MASC RADS SIQ-Jr	Suicidal event was significantly associated with high suicidal ideation levels at baseline (OR 2.0, 95% CI 1.1–3.8; *p* = 0.03) and elevated depressive symptomatology at baseline (OR 2.0, 95% CI 1.0–3.9; *p* = 0.04)
Black et al. [23]	*N* = 2389 Age: < 25 yr. IC: presenting to Emergency Department with injuries	Retrospective study	Records from the Canadian Hospitals Injury Reporting and Prevention Program Database about the circumstances of the injury	37.5% of self-harm injuries related to alcohol, involved the consumption of alcohol along with other drugs
Goldston et al. [4]	*N* = 180 Age: 12–19 yr. IC: discharge from an inpatient unit	Prospective, naturalistic study	FISA ISCA Lethality of Suicide Attempt Rating Scale	Increasing risk for SA as a function of increasing number of disorders (*b* = 0.90, SE = 0.08, *χ*^2^ = 141.97, HR = 2.46, *p* < 0.0001) Relationship between specific contemporaneous psychiatric disorders and SA: major depressive disorder [HR 5.53 (3.35, 9.12), *p* < 0.001], dysthymic disorder [HR 2.00 (0.99, 4.01), *p* = 0.047], depressive disorder NOS [HR 2.51 (0.77, 8.17), *p* = 0.119], generalized anxiety disorder [HR 1.96 (0.69, 5.53), *p* = 0.200], phobias [HR 1.07 (0.22, 5.31), *p* = 0.931], panic disorder [HR 2.35 (1.08, 5.16), *p* = 0.027], ADHD [HR 1.52 (0.77, 3.00), *p* = 0.216], OCC [HR 0.997 (0.33, 3.00), *p* = 0.996], CD [HR 2.31 (1.32, 4.06), *p* = 0.003], substance use disorder [HR 1.62 (0.85, 3.06), *p* = 0.134]
Asarnow et al. [11]	*N* = 210 Age: 10–18 yr. IC: suicide attempt and/or ideation	Cross-sectional study	CBCL CBQ CES-D Life Events Scale YRBS	Risk factors for SA: severe depressive symptoms (OR [95% CI] 1.03 [1.00–1.05]; *p* < 0.05), externalizing behaviour (OR [95% CI] 1.04 [1.01–1.07]; *p* < 0.01), thought problems (OR [95% CI] 1.04 [1.01–1.06]; *p* < 0.01), substance use (OR [95% CI]: 2.88 [1.43–5.79]; *p* < 0.01)
Fisher and le Grange [24]	*N* = 80 Age: mean 16.1 yr. (SD: 1.6) IC: bulimia nervosa, outpatient	Cross-sectional study	EDE K-SADS	SA not related to comorbid psychiatric diagnosis (*χ*^2^ = 0.66, *p* < 0.41) among subjects with bulimia nervosa
Goldstein et al. [16]	*N* = 405 Age: 7–17 yr. IC: bipolar disorder	Cross-sectional study	K-SADS	Risk factors for SA: psychiatric hospitalizations (OR 2.47, 95% CI 1.48–4.13, *p* = 0.001), history of self-injurious behaviour (OR 2.24, 95% CI 1.39–3.63, *p* = 0.001), mixed episodes (OR 2.03, 95% CI 1.21–3.41, *p* = 0.007), comorbid panic disorder (OR 4.0, 95% CI 1.36–11.76, *p* = 0.01), comorbid substance use disorder (OR 2.76, 95% CI 1.21–6.28, *p* = 0.02), and psychosis (OR 1.73, 95% CI 1.05–2.85, *p* = 0.03)
Weiner et al. [21]	*N* = 564 Children and adolescents IC: residential treatment and state custody	Retrospective study	Chart review discharge placements	Substance use disorders increase the risk for SA (girls: *χ*^2^ = 10.13; *p* < 0.05; boys: *χ*^2^ = 4.56; *p* < 0.01)
Storch et al. [30]	*N* = 102 Age: 7–16 yr. IC: youth with ASD diagnoses and co-occurring anxiety problems	Cross-sectional study	ADIS CBCL CIS-PV MASC PARS	Twenty percent of the whole sample (20/102) endorsed either thinking a lot about death or dying, having suicidal thoughts, or having a history of a suicide attempt The presence of a comorbid diagnosis of major depressive disorder/dysthymia and post-traumatic stress disorder significantly increases the likelihood of displaying suicidal thoughts and behaviours
Czyz et al. [31]	*N* = 373 Age: 13–17 yr. IC: suicide attempters or ideators in previous month	Prospective study (9 months)	BHS CDRS-R PEPSS PESQ SIQ-Jr YSR	Rehospitalisation significantly increased the risk of post discharge suicide attempts during follow-up period (hazard ratio = 3.13, *p* < 0.001)

*ADHD* attention deficit/hyperactivity disorder; *ADIS* anxiety disorder interview schedule-child and parent versions, *ADS* Adolescent Depression Scale, *ASD* autism spectrum disorder, *BDI* Beck Depression Inventory, *BHS* Beck Hopelessness Scale, *CBCL* child behavior checklist, *CBQ* Conflict Behavior Questionnaire, *C-CASA* Columbia Classification Algorithm of Suicide Assessment, *CD* conduct disorder, *CDRS-R* Child Depression Rating Scale-Revised, *CES-D* Center for Epidemiological Studies of Depression, *CI* confidence interval; *CIS-PV* Columbia Impairment Scale-Parent Version, *CGI-S* Clinical Global Impression-Severity Subscale, *EDE* eating disorder examination, *EDI-2* Eating Disorder Inventory, *FISA* follow-up interview schedule for adults, *IC* inclusion criteria, *ISCA* interview schedule for children and adolescents, *K-SADS* kiddie-schedule for affective disorders and schizophrenia, *MASC* Multidimensional Anxiety Scale for Children, *ODD* oppositional defiant disorder, *OR* odds ratio, *PARS* Pediatric Anxiety Rating Scale; *PESQ* Personal Experience Screening Questionnaire; *PEPSS* Perceived Emotional/Personal Support Scale, *RADS* Reynolds Adolescent Depression Scale, *SA* suicide attempt, *SIAB-EX* structured interview for anorexic and bulimic disorders, *SIQ-Jr* suicidal ideation questionnaire adapted for adolescents, *yr.* years; *YRBS* youth risk behavior survey, *YSR* youth self report

**Table 2:** Psychological factors. Non-clinical samples

References	Sample	Type of study	Measures	Results
Singareddy et al. [28]	*N* = 693 Age = 5–12 yr. IC: students	Cross-sectional study	CBCL 4-point Likert scale measured suicidal behaviour polysomnogram	Higher percent of REM sleep in subjects with self-harm behaviours (*p* = 0.045), even after adjusting for demographics and depression
Kelleher et al. [27]	*N* = 1112 Age: 13–16 yr. IC: students	Prospective cohort study	Adolescent psychotic Symptoms Screener Paykel Suicide Scale SDQ	Among adolescents who reported psychotic symptoms, 14% reported a SA by 3 months (OR 17.91; 95% CI 3.61–88.82) and 34% by 12 months (OR 32.67; 95% CI 10.42–102.41). OR acute SA: 67.50 (95% CI 11.41–399.21)
O’Connor et al. [20]	*N* = 2008 Age: 15–16 yr. IC: students	Cross-sectional survey	Version of the CASE questionnaire	Factors independently associated with self-harm Girls: smoking (OR range 2.06–2.36 according to number of cigarettes; *p* < 0.05), drug use (OR 1.95; 95% CI 1.19–3.18; *p* < 0.01), and anxiety (OR 1.13; 95% CI 1.06–1.19; *p* < 0.001) Boys: smoking (OR range 11.0–7.74 according to number of cigarettes; *p* < 0.001) and anxiety (OR 1.17; 95% CI 1.07–1.27; *p* < 0.001)
Arria et al. [5]	*N* = 1249 Age: 17–19 yr. IC: first-year college students	Prospective cohort study	BDI DI QRI SSAS	Suicidal ideation among individuals without high levels of depressive symptoms was predicted by: affective dysregulation (*χ*^2^ 18.6; OR 1.1; 95% CI 1.0–1.1), and alcohol use disorder (*χ*^2^ 7.9; OR 2.0; 95% CI 1.2–3.3; *p* < 0.01)
Rossow et al. [19]	*N* = 30532 Age: 15–16 yr. IC: students	Cross-sectional international survey	Self-administered questionnaires	Elevated risk of deliberate self-harm among heavy drinkers (ORs between 1.7 and 4.2; *p* < 0.05)
Spann et al. [9]	*N* = 176 Age: 13–19 yr. IC: students	Cross-sectional study	HSC RADS RCS SEQ	When controlling for depression, no significant relationship between hopelessness and suicidal ideation [*B* = − 0.051, *F*(2, 167) = 0.422, *p* = 0.52] or attempt [*B* = − 0.04, *F*(2, 172) = 0.20, *p* = 0.66]
Park et al. [13]	*N* = 501 Age: adolescents IC: students	Cross-sectional study	PACI SCL-90-R SSI	Males: life satisfaction, depression, and family communication explained 28% of the variance. Life satisfaction was the strongest predictor of suicidal ideation (*β* = − 0.315, *p* < 0.001), followed by depression (*β* = 0.247, *p* < 0.001) Females: depression, smoking, and life satisfaction explained 38% of the variance. Depression was the strongest predictor of suicidal ideation (*β* = 0.375, *p* < 0.001), followed by smoking (*β* = − 0.265, *p* < 0.001)
Wilcox et Anthony [22]	*N* = 169 Age at first assessment: 8–15 yr. IC: students	Prospective cohort study	Self-administered standardized questions	Early-onset (< 16 yr.) of cannabis use increased risk of SA (cannabis-associated RR = 1.9; *p* = 0.04) and suicide ideation in females (RR = 2.9; *p* = 0.006). No association for early-onset alcohol and tobacco use
Beautrais [14]	*N* = 60 suicide completers (age: 14–24 yr.); 125 medically serious SA (age: 13–24 yr.), and 151 non-suicidal community comparison subjects (age: 18–24 yr.)	Cross-sectional study	Semi-structured interview Threatening life experiences	Suicide attempters group vs non-suicidal subjects Male gender (OR 9.9, 95% CI 3.5–28.0, *p* < 0.0001), lack of formal educational qualification (OR 7.0, 95% CI 2.8–17.7, *p* < 0.0001), mood disorder in the preceding month (OR 4.4, 95% CI 1.4–14.0, *p* < 0.05), history of psychiatric care (OR 2.6, 95% CI 1.04–6.8, *p* < 0.05), and exposure to recent stressful life events (OR 13.8, 95% CI 4.6–40.8, *p* < 0.0001) SA vs non-suicidal subjects: lack of formal educational qualification (OR 6.0, 95% CI 2.6–13.9, *p* < 0.0001), mood disorder in the preceding month (OR 17.1, 95% CI 7.0–41.5, *p* < 0.0001), history of psychiatric care (OR 2.7, 95% CI 1.2–6.0, *p* < 0.05), and exposure to recent stressful life events (OR 8.4, 95% CI 3.3–20.9, *p* < 0.0001) Fatal vs non-fatal suicide attempt: male gender [OR 3.7, 95% CI 1.7–8.2, *p* < 0.001)], and mood disorder in the preceding month (OR 4.3, 95% CI 2.1–8.7, *p* < 0.0001)
Agerbo et al. [25]	*N* = 496 suicide victims and 24,800 matched controls Age: 10–21 yr.	Cross-sectional study	Data from longitudinal Danish registers	The strongest risk factor for suicide completion was mental illness in the young (attributable risk 15%) (95% CI 12–17): schizophrenia (IRR 33.1, 95% CI 16.5–66.3), affective disorders (IRR 24.3, 95% CI 6.64–88.7), eating disorders (IRR 84.9, 95% CI 7.17–1006), and other diagnoses (IRR 10.8, 95% CI 7.75–15.0)
King et al. [8]	*N* = 1285 Age 9–17 yr. IC: NIMH Methods for the Epidemiology of Child and Adolescent Mental Disorders (MECA) Study	Cross-sectional study	MECA Service Utilization and Risk Factors Instruments	Controlling for demographics: current mood (OR 11.4; 95% CI 6.9–19.0) or anxiety disorder (OR 6.1; 95% CI 3.9–9.5), ever having smoked marijuana (OR 3.1; 95% CI 1.6–5.9), becoming drunk in the past 6 months (OR 3.4; 95% CI 1.9–6.1), currently smoking > 1 cigarette/day (OR 4.3; 95% CI 2.1–8.7) Adjusting for mood, anxiety, or disruptive disorder: becoming drunk in the past 6 months (OR 2.1; 95% CI 1.1–4.1), currently smoking > 1 cigarette/day (OR 2.3; 95% CI 1.0–5.2)
Hultén et al. [15]	*N* = 1264 Age: 15–19 yr. IC: SA	Longitudinal study	WHO/EURO Multicentre Study on Suicidal Behaviour	Repetition more frequent among individuals who had used a “hard” versus a “soft” method (OR 1.51, 95% CI 1.11–2.05). Previous SA was an independent predictor of repetition (OR 3.21, 95% CI 2.35–4.40)
McKeown et al. [17]	*N* = 359 IC: students	Longitudinal study	CES-D Coddington Life Events Scale for Adolescents FACES-II K-SADS	Impulsivity was a significant predictor of suicidal plans (OR 2.26; 95% CI 1.27–4.02) but not of suicidal ideation or attempts Prior suicidal behaviour was associated with suicidal plans (OR 10.63; 95% CI 1.95–57.95)
Sourander et al. [29]	*N* = 5302 Age: 8 yr. at assessment. Follow-up data recorded until age of 25 yr. IC: birth cohort study	Prospective population-based study	CDI Rutter Questionnaire Death certificates Finnish Hospital Discharge Register Finnish Cause of Death Register	Among males, completed or serious SA was predicted at the age of 8 yr. by Rutter parent total score (OR 7.7; 95% CI 3.6–16.6; *p* < 0.001), Rutter teacher total score (OR 5.6; 95% CI 2.6–12.0; *p* < 0.001), psychological problems as reported by the primary teacher (OR 2.8; 95% CI 1.2–6.2; *p* < 0.01), conduct (OR 5.4; 95% CI 2.4–11.8; *p* < 0.001), hyperkinetic (OR 4.3; 95% CI 1.9–10.0; *p* < 0.001), and emotional (OR 4.3; 95% CI 1.9–9.4; *p* < 0.001) problems. Self-reports of depressive symptoms at the age of 8 yr. did not predict suicidal outcome

*BDI* Beck Depression Inventory, *CASE* Child and Adolescent Self Harm in Europe, *CBCL* child behavior checklist, *CES-D* Center for Epidemiological Studies of Depression, *CDI* Children´s Depression Inventory, *DI* Dysregulation Inventory, *FACES-II* Family Adaptability and Cohesion Evaluation Scales, *HSC* Hopelessness Scale for Children, *IC* inclusion criteria, *IRR* incidence rate ratio, *K-SADS* kiddie-schedule for affective disorders and schizophrenia, *MECA* methods for the epidemiology of child and adolescent mental disorders, *OR* odds ratio, *PACI* Pre-Adolescent Clinical Inventory, *QRI* Quality of Relationship Inventory, *RADS* Reynolds Adolescent Depression Scale, *RCS* Religious Coping Scale, *SA* suicide attempt, *SDQ* Strength and Difficulties Questionnaire, *SEQ* Suicide Experience Questionnaire, *SSAS* Social Support Appraisals Scale, *SSI* Scale for Suicidal Ideation, *SCL-90-R* Symptom Checklist-90-R, *yr.* years

#### Depression

Depression is considered a major factor in the aetiology of suicidality in children and adolescents [4, 8–12], and it has been reported in both clinical and non-clinical samples. Major depressive disorder was associated with a fivefold higher risk for suicide attempts, even after controlling for other disorders [4], gender, age, race, and socioeconomic status [8, 13]. In addition, results from a cross-sectional study conducted by Spann et al. suggest that depressive symptomatology (measured by means of the Beck Depression Inventory) mediate the relationship between hopelessness and suicidal behaviours [9].

Nevertheless, non-depressed adolescents may also report suicidal ideation and/or display suicidal behaviours [5, 14].

#### Previous suicide attempt

Converging results from longitudinal studies indicate that a previous suicide attempt is an important predictor of a future suicide attempt, reported in both clinical and non-clinical samples, increasing the risk more than threefold during follow-up [15, 16]. Similarly, results from other prospective studies have shown that prior suicidal behaviour is strongly associated with suicide plans [17], and a previous history of non-suicidal self-injury may predict the occurrence of future non-suicidal self-injury [18].

#### Drug and alcohol misuse

Cross-sectional and longitudinal studies evaluating alcohol consumption among adolescents have consistently shown that alcohol misuse is a risk factor for suicidal behaviour in clinical and non-clinical samples [5, 8, 18, 19]. Furthermore, alcohol misuse may trigger suicidal ideation even in the absence of high levels of depressive symptoms [5].

Relatedly, smoking and abuse of drugs (such as cannabis) may increase the risk of suicidal behaviour [8, 11, 13, 20–22], and the risk increases even more when drugs are used simultaneously with alcohol [4], which occurs quite frequently [23].

#### Other psychiatric diagnoses

Suicidal behaviour in children and adolescents may occur in relation to other psychiatric disorders, such as anxiety disorders [8, 20], eating disorders [24–26], bipolar disorder [16], psychotic disorders [25, 27], affective dysregulation [5], sleep disturbances [28], and externalizing disorders [29]. A growing interest has focused on the study of suicidal behaviour in autism spectrum disorders [30]. Risk for suicidality seems to be increased as a function of the number of comorbid disorders [4]. In addition, as illustrated in a follow-up study, rehospitalisation appears to be a strong indicator of a future risk of a suicide attempt [31].

#### Other risk behaviours

Suicidality in this age range may be associated with low instrumental and social competence, and having been in a fight in which there was punching or kicking in the previous year [8].

### Adverse life events

Serious adverse life events have been reported as preceding some suicides and/or suicide attempts [8, 14, 32]. They are rarely a sufficient cause for suicide/suicide attempts in isolation, and their importance lies in their action as precipitating factors in young people who are at risk by virtue of, e.g. a psychiatric condition and/or of other risk factors for suicidality as detailed below. In this vein, stress-diathesis models proposed that stressful life events interact with vulnerability factors to increase the probability of suicidal behaviour. Nevertheless, stressful life events vary with age. In children and adolescents, life events preceding suicidal behaviour are usually family conflicts, academic stressors (including bullying or exam stress), trauma and other stressful live events. In this review, 11 studies assessed stressors that occur before suicidal behaviour, with similar results for both studies using clinical and non-clinical samples (see Tables 3 and 4).

**Table 3:** Adverse life events. Clinical samples

References	Sample	Type of study	Measures	Results
Brent et al. [18]	*N* = 334 Age: 12–18 yr. IC: CDRS-R ≥ 40 and CGI-S ≥ 4	Prospective study	BDI BHS CBQ CDRS-R K-SADS-PL SIQ-Jr	Family conflict is a predictor of suicidal adverse event (OR 1.1, 95% CI 1.03–1.16)
Vitiello et al. [12]	*N* = 439 Age 12–17 yr. IC: Major depressive disorder	Prospective study	ADS BHS C-CASA CDRS-R K-SADS-PL MASC RADS SIQ-Jr	An acute interpersonal conflict identified in 72.7% of cases of subjects with a suicidal adverse event (84% youth–parent conflict, 16% youth–peer conflict). Identifiable recent legal problem present in 13% of those subjects with a suicidal adverse event during follow-up
Qin et al. [42]	*N* = 4160 SA; 79 completed suicides; 2370 matched controls Age: 11–17 yr.	Prospective study	Danish longitudinal population registries	Attempted and completed suicide risk significantly increased with increasing changes of residence
Asarnow et al. [11]	*N* = 210 Age: 10–18 yr. IC: SA and/or ideation.	Cross-sectional study	CBCL CBQ CES-D YRBS Life Events Scale	Stressors associated with increased SA risk Females: romantic breakups (OR 3.16; 95% CI 1.65–6.06; *p* < 0.001) and exposure to suicide/SA (OR 3.05; 95% CI 1.54–6.04; *p* < 0.001) Males: romantic breakups (OR 5.12: 95% CI 1.61–16.24; *p* < 0.01)
Kerr et al. [34]	*N* = 220 Age: 12–18 yr. IC: inpatients	Cross-sectional study	BHS PEPSS PESQ RADS SIQ-JR SSB	Suicidal ideation associated with perceptions of lower family support among females (*β* = − 0.26, *p* = 0.002, and higher peer support among males (*β* = 0.24, *p* = 0.016)

Clinical samples

*ADS* Adolescent Depression Scale, *BDI* Beck Depression Inventory, *BHS* Beck Hopelessness Scale, *CBCL* child behavior checklist, *CBQ* Conflict Behavior Questionnaire, *C-CASA* columbia classification algorithm of suicide assessment, *CDRS-R* Child Depression Rating Scale-Revised, *CES-D* Center for Epidemiological Studies of Depression, *CGI-S* Clinical Global Impression-Severity Subscale, *CI* confidence interval, *IC* inclusion criteria, *K-SADS* kiddie-schedule for affective disorders and schizophrenia, *MASC* Multidimensional Anxiety Scale for Children, *OR* odds ratio, *PEPSS* Perceived Emotional/Personal Support Scale, *PESQ* Personal Experience Screening Questionnaire, *RADS* Reynolds Adolescent Depression Scale, *SA* suicide attempt, *SIQ-Jr* Suicidal Ideation Questionnaire adapted for adolescents, *SSB* Spectrum of Suicide Behavior Scale, *yr.* years, *YRBS* youth risk behavior survey

**Table 4:** Adverse life events. Non-clinical samples

References	Sample	Type of study	Measures	Results
Wan et al. [44]	*N* = 14211 Age: mean 15.1 yr. IC: students	Cross-sectional school survey	Parent–Child Conflict Tactics Scale MSQA Screening Questionnaire	Students’ exposure to childhood abuse (physical, emotional or sexual) was significantly associated to non-suicidal self-injury behaviours (OR between 2.43 and 4.95)
Kiss et al. [45]	*N* = 387 Age: 10–17 yr. IC: post trafficking services admission	Cross-sectional study	Hopkins symptoms checklist Screening Questionnaire Harvard Trauma Questionnaire	Trafficking experiences associated with suicidal ideation: severe physical violence (AOR 3.68; 95% CI 1.77–7.67), sexual violence (AOR 3.43; 95% CI 1.80–6.54), extremely excessive work hours (AOR 2.69; 95% CI 1.38–5.26), restricted freedom (AOR 2.44; 95% CI 1.34–4.44), and threats by trafficker (AOR 3.59; 95% CI 1.92–6.73)
Pan and Spittal [32]	*N* = 8182 IC: students	Cross-sectional study	Global School-Based Health Survey	Association between suicidal ideation and religious bullying victimisation (AOR: 4.58, 95% CI 1.4–15.01) and racial bullying victimisation (AOR: 2.12, 95% CI 1.15–3.93)
Fisher et al. [40]	*N* = 2141 Age: 12 yr. IC: population-based birth cohort	Longitudinal study	Structured interview CDI MASC WISC-IV	Association between exposure to frequent bullying by peers before age 12 and self-harm at 12 yr., even after controlling for lifetime exposure to physical maltreatment by adults, internalising and externalizing problems at age 5, and IQ at age 5 (bullying victimisation reported by mother: RR 1.92, 95% CI 1.18–3.12; (bullying victimisation reported by child RR 2.44, 95% CI 1.36–4.40)
Klomek et al. [39]	*N* = 5813 Age: 8 yr. IC: population-based birth cohort	Prospective study	CDI Rutter Scale Finland’s Cause of Death Registry Finnish Hospital Discharge Register	Adjusting for conduct symptoms and depression at age 8 yr., association between frequent victimisation and suicidal behaviour among girls (OR 5.2; 95% CI 1.4–19.6; *p* < 0.05)
O’Connor et al. [43]	*N* = 2008 Age: 15–16 yr. IC: students	Prospective study	Version of the CASE questionnaire	Worries about sexual orientation (OR 4.82, 95% CI 1.25–18.52, *p* = 0.022), history of sexual abuse (OR 5.26, 95% CI 1.01–27.48, *p* = 0.049), family Deliberate Self Harm (OR 4.75, 95% CI 1.46–15.47, *p* = 0.010), anxiety (OR 1.30, 95% CI 1.06–1.59, *p* = 0.011) and self-esteem (OR 0.82, 95% CI 0.69–0.98, *p* = 0.033) were associated with repeat DSH during the 6-month follow-up period Sexual abuse was the only predictive factor for first-time DSH (OR 7.19, 95% CI 1.18–43.96, *p* = 0.033)
Herba et al. [41]	*N* = 1526 Age: mean 12.29 yr. IC: population-based cohort	Prospective study	Peer nomination Youth self-report	Compared to children uninvolved in bullying, bully-victims (*p* = 0.39) and victims (*p* = 0.85) did not report increased levels of suicide ideation. Victims of bullying without parental internalising disorders were similar to those uninvolved in bullying to report suicide ideation (OR 1). Victims with rejection at home reached OR for suicide ideation close to 8
Martin et al. [37]	*N* = 2603 Age: 13 yr. (T1), 14 yr. (T2), and 15 yr. (T3). IC: students	Prospective study	A single-item measure of perceived academic performance	Cross-sectional analysis: holding locus of control and self-esteem constant, a student who perceives their academic performance as “failing” is more likely to report suicide thoughts (OR between 1.58 and 1.91), plans (OR between 1.91 and 2.15), threats (OR between 1.65 and 1.86), deliberate self-injury (OR between 1.53 and 2.15), or SA (OR between 2.56 and 3.29). Longitudinal analysis: perceived academic performance at T1 is not a significant predictor of any suicide variables at T2 or T3, except for a weak association with suicide threats at T2 (OR 1.87, 95% CI 1.03–3.40, *p* < 0.05)
Wild et al. [35]	*N* = 2946 Age: 12–26 yr. IC: students	Cross-sectional study	BDI SEQ Self-administered questionnaire	Factors associated with SA and ideation: high depression scores (ideation vs none: RRR 2.85, 95% CI 1.89–4.31, *p* < 0.001; attempt vs none: RRR 3.77, 95% CI 1.95–7.30, *p* < 0.001), and low family self-esteem scores (ideation vs none: RRR 1.47, 95% CI 1.04–2.07, *p* < 0.05; attempt vs none: RRR 3.68, 95% CI 1.87–7.23, *p* < 0.001) Low family self-esteem differentiated SA from ideation (RRR 2.50, *p* = 0.02)
Agerbo et al. [25]	*N* = 496 suicide victims and 24,800 matched controls Age: 10–21 yr.	Cross-sectional study	Data from longitudinal Danish registers	Associated parental factors: parental suicide (father: IRR^11^ 2.30, 95% CI 1.10–4.80; mother: IRR 4.75, 95% CI 2.10–10.8), admission for a mental illness (father: IRR 1.56, 95% CI 1.12–2.19; mother: IRR 1.73, 95% CI 1.29–2.32), the loss of a mother due to other causes of death (IRR 2.06, 95% CI 102–4.19) or emigration (IRR 2.09, 95% CI 1.11–3.96)
King et al. [8]	*N* = 1285 Age 9–17 yr. IC: NIMH Methods for the Epidemiology of Child and Adolescent Mental Disorders Study	Cross-sectional study	MECA Service Utilization and Risk Factors Instrument	More stressful life events in SA than ideation (*p* < 0.05) Adjusting for demographics and the presence of a mood, anxiety, or disruptive disorder Family environment: Poor vs good (OR 2.0; 95% CI 1.2–3.4), fair vs good (OR 1.3; 95% CI 0.7–2.3) Physical discipline: some vs none (OR 1.2; 95% CI 0.6–2.0) Primary caretaker: no spouse vs spouse (OR 0.7; 95% CI 0.4–1.3) Parental monitoring: low vs high (OR 3.0; 95% CI 1.3–7.0), middle vs high (OR 2.4; 95% CI 1.1–5.3) Family history of psychiatric disorder (OR 1.2; 95% CI 0.7–2.2)
McKeown et al. [17]	*N* = 359 IC: students	Prospective study	K-SADS CES-D FACES-II Coddington Life Events Scale for Adolescents	Family cohesion protects from SA (OR 0.90; 95% CI 0.86–0.95), though not from plans (OR 0.99; 95% CI 0.93–1.04) or ideation (OR 1.00; 95% CI 0.95–1.05) Undesirable life events predict suicidal plans (OR 1.09; 95% CI 1.01–1.18), but not suicidal ideation (OR 1.06; 95% CI 0.96–1.17) and attempts (OR 1.03; 95% CI 0.88–1.21)
Wagner et al. [33]	*N* = 1050 (147 SA; 261 depressed/suicidal ideators; 642 controls) Age 12–21 yr.	Cross-sectional study	Inventory of daily stresses Self-administered Questionnaire	Factors related to SA: stresses related to parents, lack of adult support outside of the home, problems with police, physical harm by a parent, running away from home, living apart from both parents, knowing someone who had completed suicide
Sourander et al. [29]	*N* = 5302 Age: 8 yr. at assessment Follow-up data recorded until age of 25 yr. IC: birth cohort study	Longitudinal study	Self-administered Questionnaire Finnish Hospital Discharge Register Finnish Cause of Death Register	Among males, completed or serious SA predicted at the age of 8 yr. by living in a non-intact family (OR 3.8; 95% CI 1.7–8.2; *p* < 0.001)

*AOR* adjusted odds ratio, *BDI* Beck Depression Inventory, *CASE* Child and Adolescent Self Harm in Europe, *CDI* Children’s Depression Scale, CES-D Center for Epidemiological Studies of Depression, *CI* confidence interval, *DSH* deliberate self-harm, *FACES-II* Family Adaptability and Cohesion Evaluation Scales, *IC* inclusion criteria, *IQ* intelligence quotient, *IRR* incidence rate ratio, K-SADS kiddie-schedule for affective disorders and schizophrenia, *MASC* Multidimensional Anxiety Scale for Children, *MECA* methods for the epidemiology of child and adolescent mental disorders, *MSQA* Multidimensional Sub-health Questionnaire of Adolescents, *OR* odds ratio, *RR* relative risk, *RRR* relative risk ratio, *SA* suicide attempt, *SEQ* Self-Esteem Questionnaire, *WISC-IV* Wechsler intelligence scale for children, fourth edition, *yr.* years

#### Family conflicts

Family conflict has been associated with suicidal behaviour [18], even after controlling for gender, age, and psychiatric disorders [8]. Adolescents with a history of a suicide attempt more frequently than controls report stress related to parents, lack of adult support outside of the home, physical harm by a parent, running away from home, and living apart from both parents [33–35]. Other family situations associated with risk for suicidality are: parental suicidal behaviour, early death, mental illness in a relative, unemployment, low income, neglect, parental divorce, other parent loss, and family violence [20, 25, 29, 36].

#### Academic stressors

Students who perceive their academic performance as failing seem to be more likely to report suicidal thoughts, plans, threats, and attempts or deliberate self-injury [37]. Perfectionism has been reported as a personality construct that may be associated with suicidality in adult samples. However, results from a pioneering study in children and adolescents evaluating the Perfectionism Social Disconnection Model suggest that the association between perfectionism and suicidality is mediated by stressful life events (being bullied) or by other psychological features such as learned helplessness [38].

#### Trauma and other adverse life events

In addition to family conflicts or academic performance problems, early traumatic experiences and other adverse life events have been associated with suicidal behaviours. A history of childhood sexual abuse is associated with a 10.9-fold increase in the odds of a suicide attempt between the ages of 4 and 12 years and a 6.1-fold increase in the odds of an attempt between the ages of 13 and 19 years [36].

Victims of bulling have higher rates of suicidal behaviour and ideation [39, 40], and some victims may be particularly vulnerable to suicidal ideation due to parental psychopathology and feelings of rejection at home [41].

Change of residence may result in loss of a familiar environment as well as a breakdown of the social network, which may induce stress and adjustment problems, and therefore, increase the risk of suicidal behaviour [42].

Other stressful circumstances that may precede suicidal behaviour are peer conflict, legal problems, physical abuse, worries about sexual orientation, romantic breakups, exposure to suicide/suicide attempts, and physical and/or sexual violence among trafficked victims [11, 12, 20, 32, 39, 43–45].

### Temperament and character

Some personality traits have been identified as predisposing factors for suicidality. Neuroticism, perfectionism, interpersonal dependency, novelty-seeking, pessimism, low self-esteem, a perception that one is worse off than one’s peers, and self-criticism have been implicated as risk factors for suicidality in adolescents [20, 37, 46–49]. Similarly, maladaptive coping styles have been described as a risk factor for both depression and suicidal ideation [50].

Impulsivity has emerged as an important issue in suicidality [17, 20, 51, 52], with 50% of adolescents having only started thinking about self-harm less than an hour before the act itself [20] (Tables 5, 6).

**Table 5:** Temperament and character. Clinical samples

References	Sample	Type of study	Measures	Results
Mirkovic et al. [50]	*N* = 167 Age: 13–17 yr. IC: suicide attempters, inpatients	Cross-sectional study	K-SADS Adolescent Coping Scale Life Events Questionnaire Columbia-Suicide Severity Rating Scale	When adjusting for age, sex, stressful life events and depression, non-productive coping did not prove a significant risk factor for suicidality in the multivariate analysis (*β* = 0.03, SE = 0.021; *t* = 1.669, *df* = 111, *p* = 0.095)
Csorba et al. [47]	*N* = 90 Age: 14–18 yr. IC: depressive outpatients	Cross-sectional study	JTCI M.I.N.I Plus	Suicidal-depressive adolescents exhibited significantly higher novelty-seeking compared to “pure” depressive clinical peers (Mann–Whitney *U*: 665.5; *p* = 0.007)
Dougherty et al. [52]	*N* = 56 Age: 13–17 yr. IC: inpatients with a history of NSSI^3^	Cross-sectional study	BIS Lifetime Parasuicide Count II Two Choice Impulsivity Paradigm Go-Stop Paradigm	Hospitalization analyses: compared to the NSSI-only group, the NSSI + SA group had significantly higher ratings on Barratt Impulsiveness Scale (*F* = 7.68; *df* = 1.54; *p* = 0.008; observed power = 0.78; Cohen’s *d* = 0.77), and greater preference for the smaller-sooner rewards during the Two Choice Impulsivity Paradigm (*F* = 5.47; *df* = 1.54; *p* = 0.023; observed power = 0.63; Cohen’s *d* = 0.62) Follow-up analyses: the NSSI + SA group showed a significantly greater preference for the impulsive smaller-sooner choices (main effect of Group: *F* 1.26 = 6.37, *p* = 0.018; observed power = 0.68; Cohen’s *d* = 0.88)
Enns et al. [48]	*N* = 78 Age: 12–18 yr. IC: inpatients; suicidal ideation or behaviour as reason for admission	Prospective study	CAPS SIQ	Correlations between the Suicidal Ideation Questionnaire scores and personality measures: neuroticism (0.39, *p* < 0.001), self-criticism (0.38, *p* < 0.01), dependency (0.29, *p* < 0.01), self-oriented perfectionism (0.12, *p* = NS), and socially prescribed perfectionism (0.32, *p* < 0.01) Neuroticism (*B *= 0.194; Wald = 6.26; *p* = 0.01) was predictive of psychiatric readmission within 1 year
Horesh et al. [51]	*N* = 65 Age: 13–18 yr. IC: inpatients	Cross-sectional study	BDI BHS Child Suicide Potential Scale Overt Aggression Scale Impulsiveness-Control Scale	No significant differences in impulsiveness for the depressed suicidal group versus the depressed non-suicidal group [*F* (1, 30) = 1.09, *p* = 0.05] Impulsiveness and aggression correlated significantly and positively with suicidal behaviour (aggression: *r* = 0.50, *p* < 0.01; impulsiveness: *r* = 0.40, *p* < 0.05) among borderline personality disorder adolescents, but not in depressed adolescents

*BDI* Beck Depression Inventory, *BHS* Beck Hopelessness Scale, *BIS* Barratt Impulsiveness Scale, *CAPS* Child and Adolescent Perfectionism Scale, *IC* inclusion criteria, *JTCI* Junior Temperament Character Inventory, *K-SADS* kiddie-schedule for affective disorders and schizophrenia, *M.I.N.I Plus* mini international neuropsychiatric interview, *NSSI* non-suicidal self-injury, *SA* suicide attempt, *SIQ* Suicidal Ideation Questionnaire, *yr.* years

**Table 6:** Temperament and character. Non-clinical samples

References	Sample	Type of study	Measures	Results
O’Connor et al. [20]	*N* = 2008 Age: 15–16 yr. IC: pupils	Cross-sectional study	Version of the CASE questionnaire	Optimism protects girls from self-harm (OR 0.93; 95% CI^4^ 0.88–0.97; *p* < 0.005)
Chabrol and Saint-Martin [46]	*N* = 312 Age: 14–18 yr. IC: students	Cross-sectional study	CES-D Youth Psychopathic traits Inventory	Affective component of psychopathic traits is an independent predictor of suicidal ideation (*β* = 0.17, *t* = 3.04, *p* = 0.002)
Martin et al. [37]	*N* = 2603 Age: 13 yr. (time 1), 14 yr. (time 2), and 15 yr. (time 3). IC: students	Prospective study	A single-item measure of perceived academic performance Rosenberg’s Self-esteem Scale Nowicki–Strickland Locus of Control Scale for Children	Low self-esteem associated with suicide thoughts (OR between 2.39 and 3.48), plans (OR between 2.76 and 3.55), threats (OR between 2.51 and 3.72), deliberate self-injury (OR between 1.99 and 2.58), and SA^5^ (OR between 2.26 and 4.30). External attributional style associated with suicide thoughts (OR between 1.86 and 2.39), plans (OR between 1.91 and 2.74), threats (OR between 1.72 and 1.95), deliberate self-injury (OR between 2.06 and 3.34), ad SA (OR between 1.79 and 2.90)
Barber [49]	Study I *N* = 2619 Age: 11–20 yr. IC: students Study II *N* = 2111 Age: 12–17 yr. IC: students	Cross-sectional study	Study I: Structured Questionnaire. Youth suicide rate obtained from 1994 World Health Organization statistics Study II: Self-administered questionnaires	Study I: correlations between adjustment and suicide: Males: total adjustment *r*(7) = 0.74, *p* < 0.05; self-esteem *r*(7) = 0.87, *p* = 0.01; school adjustment *r*(7) = 0.81, *p* < 0.05; social adjustment NS Females: all adjustment analyses NS Study II: in males, suicidality was significantly associated with the interaction social comparison × depressed affect (*t* = 9.4, *p* < 0.001), social comparison (*t* = − 4.5, *p* < 0.001) and with the interaction social comparison × self-esteem (*t* = 9.5, *p* < 0.001). Among females, suicidality was significantly associated with depressed affect (*t* = 4.3, *p* < 0.001), the interaction social comparison × depressed affect (*t* = 5.0, *p* < 0.001), self-esteem (*t* = − 2.2, *p* < 0.05), social comparison (*t* = − 3.7, *p* < 0.001), and interaction social comparison × self-esteem (*t* = 5.2, *p* < 0.001)

*CASE* Child and Adolescent Self Harm in Europe, *CES-D* Center for Epidemiological Studies of Depression, *CI* confidence interval, *IC* inclusion criteria, *OR* odds ratio, *SA* suicide attempt, *yr.* years

## Discussion

Suicidality among children and adolescents is a topic of increasing concern, and this is reflected in the strong/large increase in the amount of literature assessing suicidality over recent years. While deaths in these populations due to other causes are decreasing, rates of suicide remain high [2]. This highlights the importance of suicidality research and a move to improving and developing suicide prevention strategies.

This review identifies several psychosocial risk factors for suicidality (Table 7).

**Table 7:** Studies investigating risk factors for suicidality among children and adolescents by type of self-injurious thought and/or behaviour

Variable	Suicide attempt	Suicidal behaviour	Suicidal ideation/plan	Non-suicidal self-injury	Self-injurious behaviour
**Clinical variables**					
Depression	[4, 8, 9, 11–14, 29, 35]	[9, 12]	[5, 9, 12–14, 35]		[12]
Previous suicide attempt	[15, 16]		[17]		
Previous suicidal ideation	[18]	[12, 18]	[18]	[18]	[12, 18]
Alcohol and substance use	[21]	[2, 4, 5, 8, 11, 13, 18–20, 22]	[5]		[23]
Eating disorders		[26]	[26]		[26]
Psychiatric disorders	[4, 8, 27]		[30]		[20]
Hospitalization	[16]				
Sleep disturbances					[20]
**Adverse life events**					
Family conflicts	[8, 12, 17, 18]	[12, 18]	[8, 12, 17, 18, 34]		[12, 18]
Interpersonal and legal problems	[12]	[12]	[12]		[12]
Change of residence	[42]				
Romantic break-up	[11]				
Exposure to suicidal behaviour	[11, 29]				
Bullying		[39]	[32, 41, 44]		[40]
Abuse			[45]		[43]
Sexual orientation					[43]
Academic performance			[37]		
**Temperament and character**					
Novelty seeking	[47]				
Impulsiveness	[4, 52]	[52]	[17]	[52]	
Neuroticism, pessimism, perfectionism, dependence			[48]		[20]
Low self esteem	[37]		[37]		[37]
External attributional style	[37]		[37]		[37]

The majority of publications reviewed in this present work indicate that young people with suicidal behaviour had significant psychiatric problems, mainly depressive disorders and substance abuse disorders. The presence of a major depressive disorder increases the risk of suicide attempts [4]. Nevertheless, mood disorders do not explain all suicidal ideation and behaviours [5], and important distinctions must exist between depressed adolescents who have experienced suicidal ideation but have never attempted suicide and those who have done so. The evidence clearly highlights the complexity of suicidality and points towards an interaction of factors contributing to suicidal behaviour. Previous history of suicide attempts can identify a population at risk [15, 17], as does the concurrence of different disorders [4].

However, predicting which adolescents are likely to repeat their suicidal behaviour is still an area that needs further development. The natural history of suicidal behaviour among children and adolescents is not completely delineated. Clearly, more information is needed to understand the complex relationship between risk factors for suicidality and to be able to establish prevention strategies for suicidality in children and adolescents. Prospective studies with adequate sample sizes are needed to investigate these multiple variables of risk concurrently and over time.

Drug and/or alcohol misuse may also increase the risk for suicide attempt [8, 11, 18]. Acute intoxication may even trigger the suicidal act in vulnerable individuals by increasing impulsiveness, enhancing depressive thoughts and suicidal ideation, limiting cognitive functions and ability to see alternative coping strategies, and reducing barriers to self-inflicted harm [53]. In this vein, drug and/or alcohol misuse may act as proximal but also distal risk factors for suicidality and also may mediate or moderate the influence of other risk factors on suicidality [54]. Moreover, common neurobiological vulnerability has been described in depression, impulsivity and drug and/or alcohol use disorders such as a greater serotonergic impairment [53], which may help explain their frequent co-association and also their relationship with suicidal behaviour, a violent behaviour associated with disturbances in the serotonergic system [53].

In addition, vulnerability to suicidal behaviour may be, at least to some degree, mediated by some personality traits, such as neuroticism and impulsivity [17, 20, 48, 51, 52]. The association of poor emotional regulation strategies and behavioural impulsivity with suicidal behaviour leads to consider the existence of affective regulation vulnerability among children and adolescents at risk for suicidality.

Stressful life events may act as precipitating factors for suicidal behaviour. Our review identified several circumstances, such as family problems and peer conflicts that may exceed the coping strategies of some adolescents [8, 18, 20, 25, 29, 33–36]. Nevertheless, it is important to note that some investigations suggest that it is the accumulation of stressful life events, and not the presence of one isolated stressful life event that appears to be related to later suicidal behaviours [55]. However, as not all children exposed to stressful life events develop suicidal behaviours, some authors state that suicidality is not simply a logical response to extreme stress [54], which in turn leads to the hypothesis of a stress diathesis model of suicidal behaviour [56]. Thus, from a suicidal behaviour prevention standpoint, further investigation is needed to clarify the relationship between stressful life events and suicidality in the paediatric population.

### Limitations

The conclusions that can be made regarding the strength of association between the risk factors presented in this review and suicidality are limited due to the relatively small amount of prospective studies that have been conducted to date [4, 5, 12, 15, 17, 18, 22, 27, 29, 31, 37, 39, 40, 43, 48]. In addition, the majority of clinical studies used/studied/observed small populations. Publication bias is likely to be present as studies reporting no association between a risk factor and suicidal behaviour may not have been published. Suicidality was not measured by means of the same instrument across all the studies. Similarly, different instruments were used to measure psychopathology or to determine other psychosocial variables, which is another limitation. The age range of participants and sociodemographic variables differs between the different studies making direct comparisons and summaries across studies difficult/troublesome.

In conclusion, this review has pulled together relevant scientific literature addressing psychosocial risk factors for suicidality in children and adolescents. It suggests that various components and factors may contribute to the risk/development of suicidality and suicidal behaviour in a young person, e.g. impulsivity, mood disorder, substance abuse, history of self-injury, and family and/or peer conflicts, to be considered as a cumulative/interactive process. The identifications of paediatric patients at high risk for suicidality and elements of resilience will improve preventative measure in targeted subgroups.
